# uc.77- Downregulation Promotes Colorectal Cancer Cell Proliferation by Inhibiting FBXW8-Mediated CDK4 Protein Degradation

**DOI:** 10.3389/fonc.2021.673223

**Published:** 2021-05-19

**Authors:** Zhijian Zheng, Dan Hong, Xiaodong Zhang, Yixin Chang, Ning Sun, Zhenni Lin, Hongyan Li, Shirui Huang, Ruirui Zhang, Qipeng Xie, Haishan Huang, Honglei Jin

**Affiliations:** ^1^ Zhejiang Provincial Key Laboratory of Medical Genetics, Key Laboratory of Laboratory Medicine, Ministry of Education, School of Laboratory Medicine and Life Sciences, Wenzhou Medical University, Wenzhou, China; ^2^ Department of Colorectal Anal Surgery, The First Affiliated Hospital of Wenzhou Medical University, Wenzhou, China; ^3^ Department of Clinical Laboratory, The Second Affiliated Hospital & Yuying Children’s Hospital of Wenzhou Medical University, Wenzhou, China

**Keywords:** uc.77-, miR-4676-5p, FBXW8, CDK4, colorectal cancer

## Abstract

Transcribed ultraconserved regions (T-UCRs) are a new type of long non-coding RNA, and the UCR has 481 segments longer than 200 base pairs that are 100% conserved between humans, rats, and mice. T-UCRs involved in colorectal cancer (CRC) have not been studied in detail. We performed T-UCR microarray analysis and found that uc.77- was significantly downregulated in CRC tissues and cell lines. Ectopic expression of uc.77- significantly inhibited the proliferation of CRC cells *in vitro* and the growth of xenograft tumors in nude mice *in vivo*. Mechanistic studies showed that uc.77- competed with FBXW8 mRNA for binding to microRNA (miR)-4676-5p through a competing endogenous RNA mechanism and inhibited the proliferation of CRC cells by negatively regulating CDK4. The present findings highlight the role of the uc.77-/miR-4676-5p/FBXW8 axis in CRC and identify uc.77- as a potential novel target for the treatment of CRC.

## Introduction

Colorectal cancer (CRC) is the third most common cancer worldwide. Approximately 1.93 million new cases of CRC were reported in 2020, with an incidence rate of 10.0% and a mortality rate of 9.4% (1). In China, CRC is the third most common malignant tumor and the fourth leading cause of cancer-related death (2). Elucidating the pathogenesis of CRC and identifying diagnostic markers are urgent needs.

Ultraconserved regions (UCRs) are non-coding gene sequences composed of 481 segments that are 100% conserved among mammals such as humans, rats, and mice. These segments can be located on exons or introns of the coding gene. RNAs transcribed from the UCR region are called T-UCRs or ultra-conserved (uc) RNAs. Non-coding RNAs transcribed from UCRs may regulate gene expression (3).T-UCRs are a new type of long non-coding RNAs (lncRNAs) that can function as oncogenes or tumor suppressors in different tumor types (4). uc.338 is highly expressed in liver cancer cells, and it promotes cell proliferation by affecting cell cycle progression (5). uc.338 is also expressed at high levels in CRC, and it promotes metastasis and invasion of CRC (6). On the other hand, uc.160+ is expressed at low levels in gastric cancer, where it plays a tumor suppressor role (7). The role of T-UCRs in tumor development is not well understood.

Cyclin-dependent kinase 4 (CDK4) is an important regulator of the cell cycle that forms a complex with CDK6 and cyclin D1, and phosphorylates retinoblastoma (Rb) protein. Inactivated Rb releases E2F, thereby promoting the transition from G0/G1 phase to S phase of the cell cycle (8, 9). CDK4 is frequently dysregulated in cancer, and the development of CDK4-targeted therapies is important. However, the development of CDK inhibitors is challenging, and several clinical trials have shown disappointing results (10, 11). uc.77-, a new lncRNA that was identified as a potential target for the treatment of CRC, is involved in the regulation of CDK4. In this study, we show that uc.77- competes with FBXW8 mRNA for binding to microRNA (miR)-4676-5p, thereby promoting the expression of FBXW8 and the ubiquitination of CDK4, which inhibits the proliferation of CRC cells.

## Materials and Methods

### T-UCR Microarray

This study was previously detected by our team at Kangcheng Bio (Shanghai, China). The total RNA of each sample was quantified with NanoDrop ND-1000 (Thermo Fisher Scientific), and RNA integrity was evaluated by standard denaturing agarose gel electrophoresis. For microarray analysis, the Agilent array platform (Agilent Technologies, Santa Clara, California) was employed. Sample preparation and microarray hybridization were performed according to the manufacturer’s standard protocol. In short, the purified mRNA and LncRNA were amplified by random primer method (Arraystar Flash RNA Labeling Kit, Arraystar) and transcribed into fluorescent cRNA along the full length of the transcript. The labeled cRNA was hybridized to human T-UCR array (8×60K, Arraystar). After cleaning the slides, the array was scanned by the Agilent scanner G2505C (Agilent Technologies). The extracted data was normalized using Agilent GeneSpring GX v12.1 software. Use GeneSpring GX v12.1 and a signal processing algorithm called Otsu method for further data analysis. Finally, the differentially expressed T-UCRs between the samples were identified by fold change filtering.

### Plasmids, Antibodies, and Reagents

The plasmid overexpressing uc.77- and the corresponding control plasmid were purchased from TsingKe Biological Technology (Beijing, China). The CDK4 plasmid was constructed and used in a previous study (12). Mimics for miR-4676-5p and miR-3605-5p were purchased from Ruibo Biotechnology (Guangzhou, China). The wild-type and mutant luciferase reporter genes for the predicted miR-4676-5p binding sites at uc.77- and at the FBXW8 3′ untranslated region (3′-UTR) were purchased from TsingKe Biological Technology.

Short hairpin RNAs (shRNAs) against FBXW8 and the control plasmid were purchased from Open Biosystems (Thermo Fisher Scientific, NY, USA). Antibodies against CDK2 (sc-6248), CDK4 (sc-260), CDK6 (sc-177), cyclin D1 (sc-20044), cyclin E2 (sc-481), and FBXW8 (sc-514385) were purchased from Santa Cruz Biotechnology (Santa Cruz, CA, USA). Antibodies against cyclin E2 (sc-481), FBXW11 (13149-1-AP), and HECTD3 (11487-1-AP) were purchased from Proteintech (Chicago, IL, USA). Antibody against α-tubulin (ab7291) was purchased from Abcam (Cambridge, UK). Cycloheximide (CHX) and MG132 were purchased from Calbiochem (San Diego, CA, USA).

### Cell Culture and Transfection

The human CRC cell lines HCT116, HT-29, LoVo, and SW620 were purchased from the Shanghai Chinese Academy of Sciences. SW480 and CCD-18Co cells were purchased from American Type Culture Collection. HCT116 cells were cultured in M5A medium (PM150710; Procell) supplemented with 10% fetal bovine serum (FBS; 42Q8982K; Gibco), and SW620 cells were cultured in RPMI 1640 (11875–093, Gibco) supplemented with 10% FBS. The Polyjet Transfection Reagent (SL100688, SignaGen Laboratories) was used for stable transfection. According to the different antibiotic resistance plasmids transfected, selection was performed using puromycin (4–6 μg/mL; J593; Amresco Inc.) or G418 (1000–1500 μg/mL; sc-29065;Santa Cruz, Dallas, TX, USA) for 3–4 weeks.

### Lentiviral Infection

The 293T cells were inoculated into 6-well plates in 2 mL DMEM containing 10% FBS and grown until reaching a density of 60–70%. A combination of 1.2 μg pMD2.G (12259, Addgene), 1.2 μg psPAX.2 (12260, Addgene), and 2 μg plasmid was mixed with 50 μL serum-free DMEM containing 6 μL Polyjet. The mixture was incubated at room temperature for 15 min and added to 293T cells in 6-well plates, which were placed in a 5% CO_2_ incubator at 37°C for 6–8 h. After replacing the medium with fresh DMEM containing 10% FBS, the plates were incubated for 48 h. Then, 2 mL of supernatant containing virus was collected, centrifuged at 3000 rpm for 30 min, and passed through a 0.45 μm filter with a syringe. The virus supernatant was added to the cell culture medium at a ratio of 1:1, and stable cell lines were selected with puromycin.

### Anchorage-Independent Growth

To examine the anchorage-independent growth of CRC cells, a suspension culture containing 10^4^ stably transfected HCT116 (uc.77-) or SW620 (uc.77-) cells and their control carrier cells was placed in a 6-well plate containing 0.33% agar and 10% FBS basal medium. The plates were incubated at 37°C and 5% CO_2_ for 2–3 weeks, and the number of colonies was counted under a DMi1 microscope (Leica Microsystems, Buffalo Grove, IL, USA). The result is presented as the mean ± SD of colonies/10,000 inoculated cells (13).

### Cell Proliferation

Approximately 1500 HCT116 (vector), HCT116 (uc.77-), SW620 (vector), or SW620 (uc.77-) cells were seeded into 96-well plates in 200 μL of complete medium/well. After the cells adhered to the wall, the complete medium was replaced with 0.1% FBS medium, and cells were starved for 12 h to synchronize the cycle. The medium was then replaced with fresh complete medium, and cells were incubated for the indicated number of days. The cell proliferation index was determined using the CellTiter-Glo Luminescent Cell Viability Assay Kit (G7572; Promega). Briefly, the culture medium from 96-well plates was discarded, and 25 μL PBS mixed with 25 μL of the reagents provided in the kit were added to the cells. The plates were shaken for 2 min and incubated at room temperature for 10 min. Luminescence was detected using the Centro LB 960 luminometer (Berthold, Bad Wildbad, Germany) (14, 15).

### Cell Cycle

The stably transfected HCT116 (uc.77-) and SW620 (uc.77-) cells and control vector cells seeded in 6-well plates were washed with PBS, digested with 0.25% EDTA trypsin, and then fixed in 70% ethanol at 4°C overnight. The cells were then washed with pre-cooled PBS and treated with 500 μL RNase A (KGA511; KeyGen Biotech, Nanjing, China) and propidium iodide at a ratio of 1:9. The plates were incubated at room temperature for 30–60 min and analyzed using the CytoFlex flow cytometer (Beckman Coulter, Brea, CA, USA) and CytExpert software.

### qRT-PCR

Total RNA was isolated from cells using Trizol (15596018, Invitrogen), and cDNA was synthesized using the SuperScript™ First Strand Synthesis System (18091200, Invitrogen). The qRT-PCR analysis was performed using the Q6 real-time PCR system (Thermo Fisher Scientific, Waltham, MA, USA) and the fast SYBR Green Master Mix kit (4385614, Applied Biosystems). Real-time PCR primers for uc.77- (forward, 5′-CTGTCACACTGCTCCCAAGAA-3′; reverse, 5′-GGGAGAACTCA GCCAAAGATG-3′) were purchased from Sunny Biotechnology (Shanghai, China). Other primers used in this study were synthesized by TsingKe Biological Technology, including FBXW8 (forward, 5′-CTACAGCCTGGATGAGTTCCG-3′; reverse, 5′-TGCAATCACCTTCCACGTCT-3′) and GAPDH (forward, 5′-GACTCATGACC ACAGTCCATGC-3′; reverse, 5′-CAGGTCAGGTCCACCACTGA-3′). GAPDH was used as an endogenous control.

### Western Blot Analysis

Protein was extracted from cells using boiling buffer (1% SDS, 1 mM Na3VO4, 10 mM Tris-HCl [pH 7.4]) on ice. The samples were heated at 100°C for 5 min, and nucleic acid fragments were sonicated. The protein samples were separated by polyacrylamide gel electrophoresis and transferred to a PVDF membrane (Bio-Rad, Hercules, CA, USA). The membrane was blocked with 5% skim milk in TBST for 1 h, incubated with the indicated primary antibodies at 4°C overnight, and then incubated with alkaline phosphatase-conjugated secondary antibody at 4°C for 3 h. Films were processed with ECF (RPN5787; GE Healthcare, PA, USA) for signal detection, and images were captured on the Typhoon FLA 7000 scanner (GE Healthcare) (16).

### Dual Luciferase Reporter Assay

Luciferase assays were performed using a dual luciferase reporter system (Promega) according to the manufacturer’s instructions. The wild-type and mutant uc.77- and FBXW8 3′-UTR fragments cloned into the pmirGLO vector were purchased from TsingKe Biological Technology. The wild-type or mutant FBXW8 3′-UTR vector and miR-4676-5p mimic were co-transfected with pRL-TK using the RiboFECT CP Transfection Kit (C10511-05, Ruibo Biotechnology). Cells were cultured for 48 h before addition of cell lysis buffer and incubation at room temperature for 15 min. Then, 20 μL aliquots were pipetted into a 96-well plate and treated with 40 μL Luciferase Assay Reagent II in the dark. The luciferase activity in each well was measured in a Centro LB 960 luminometer (Berthold). After adding 40 μL Stop&Glo reagent to the same well, the luciferase activity value was analyzed again.

### Xenotransplantation Model in Nude Mice

Female BALB/c athymic nude mice (3–4 weeks old) were purchased from Jiangsu Jicui Yaokang Biotechnology Co., Ltd. Animal experiments were performed in the Animal Center of Wenzhou Medical University according to the protocol approved by the Experimental Animal Ethics Committee. After 1–2 weeks of adaptive culture, 12 nude mice were randomly divided into two groups of six mice each. A volume of 100 μL HCT116 (vector) or HCT116 (uc.77-) cell suspension in PBS containing 4 × 10^6^ cells was subcutaneously injected into the right ventral side. After 3–4 weeks, the mice were sacrificed, and the tumors were surgically removed, photographed, and weighed. One half of each tumor was fixed in 4% paraformaldehyde for immunohistochemistry (IHC). The remaining half was frozen and stored at -80°C and used for RNA extraction (17).

### Protein Degradation Experiment

HCT116 (vector) and HCT116 (uc.77-) cells were seeded in 6-well plates containing complete medium. When the cell density reached 80%, the medium was replaced with fresh medium containing 0.1% FBS, and cells were starved for 12 h. Then, the medium was replaced with fresh medium containing 10 μM MG-132 and 10% FBS for 5 h. After replacing the medium with fresh medium containing 50 μg/mL CHX, the plates were placed in a 37°C incubator with 5% CO_2_. Cells were collected at 0, 3, 6, and 12 h. The CDK4 degradation rate was assessed by western blotting (18).

### RNA Antisense Purification (RAP)

Cell suspensions were prepared according to the instructions of the RAP kit (Bersinbio, Guangzhou, China). Briefly, a biotin-labeled 50 bp antisense probe (detailed information is provided in **Table S1**) was added to the lysis solution. After denaturation and hybridization, streptavidin magnetic beads were added, and the eluate was obtained after washing to remove non-specifically bound RNA. The RNA interacting with the lncRNA was collected, transcribed into cDNA, and analyzed by qRT-PCR.

### Immunohistochemistry (IHC)

Ki67 expression was detected by IHC in formalin-fixed paraffin-embedded specimens from mice using a specific primary antibody against Ki67 (ab16667, Abcam). IHC staining was performed using a kit from Boster Bio-Engineering Company (SA1022; Wuhan, China). Immunostained images were captured using the Nikon Eclipse Ni microsystem (DS-Ri2), and the integrated optical density of each stained area was calculated.

### Clinical Specimens

Approved by the Ethics Committee of Wenzhou Medical University, a total of 150 pairs of samples of human CRC tissue and corresponding adjacent normal tissues were isolated from patients in the First Affiliated Hospital of Wenzhou Medical University, which has been described in the accepted article (Frontiers in Oncology Manuscript ID: 668743). Each sample was quick-frozen in liquid nitrogen during separation. One third of each sample was used for RNA extraction, and synthesized cDNA was stored at -80°C. The remaining sample was fixed with 4% paraformaldehyde, embedded into a wax block, and stored at room temperature.

### Statistical Analysis

GraphPad Prism 7 was used for graph production and data analysis. Experimental data are expressed as the mean ± standard deviation (mean ± SD). The significance of differences between groups was determined using the Student’s t-test. Significance was accepted at P < 0.05.

## Results

### uc.77- Screening and Expression in CRC Tissues and Cells

**Figure 1:**
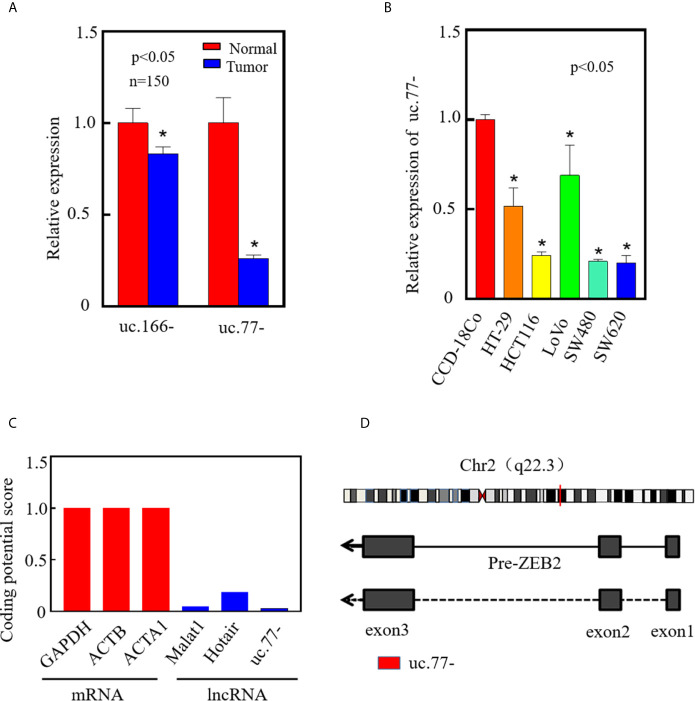
uc.77- is downregulated in human CRC tissues and cell lines. **(A)** The expression of top two downregulated T-UCRs in CRC tissues was detected by qRT-PCR (P < 0.05). **(B)** Expression of uc.77- in human colorectal cancer cell lines (HT-29, HCT116, LoVo, SW480, and SW620) and human normal colorectal epithelial cells (CCD-18Co) detected by qRT-PCR. **(C)** Coding potential score of uc.77- measured by CPC2.0. **(D)** Genome structure of uc.77- from the third exon of ZEB2 located in chromosome 2q22.3. Data are presented as the mean ± SD, *P < 0.05.

To investigate the roles T-UCRs in CRC development, we analyzed T-UCR microarray data (GSE167326) published by our group. The top two downregulated T-UCRs, uc.77- and uc.166-, were identified by real-time PCR in 150 pairs of clinical tissues (tumor/normal tissue), of which uc.77- was the most significantly downregulated transcript in CRC (**Figure 1A**). Therefore, uc.77- was selected for further analysis, and its biological functions and mechanisms were examined *in vivo* and *in vitro*. Analysis of uc.77- in CRC cell lines (HT-29, HCT116, LoVo, SW480, and SW620) showed that uc.77- expression was significantly lower in HCT116 and SW620 cells than in the human normal colon tissue cell line CCD-18Co cells (**Figure 1B**). The coding potential calculator CPC2.0 (http://cpc2.gao-lab.org/) was used to determine whether uc.77- is a non-coding RNA, we found that the coding potential of uc.77- is significantly lower than the coding genes (GAPDH;ACTB and ACTA1) and typical non-coding RNAs (Malat1 and Hotair) (**Figure 1C**) (19). The results showed that uc.77- is an exon from the Zinc Finger E-Box binding homeobox 2 (ZEB2) transcript (**Figure 1D**).

### Ectopic Overexpression of uc.77- Inhibits the Proliferation of CRC Cells *In Vivo* and *In Vitro*

**Figure 2:**
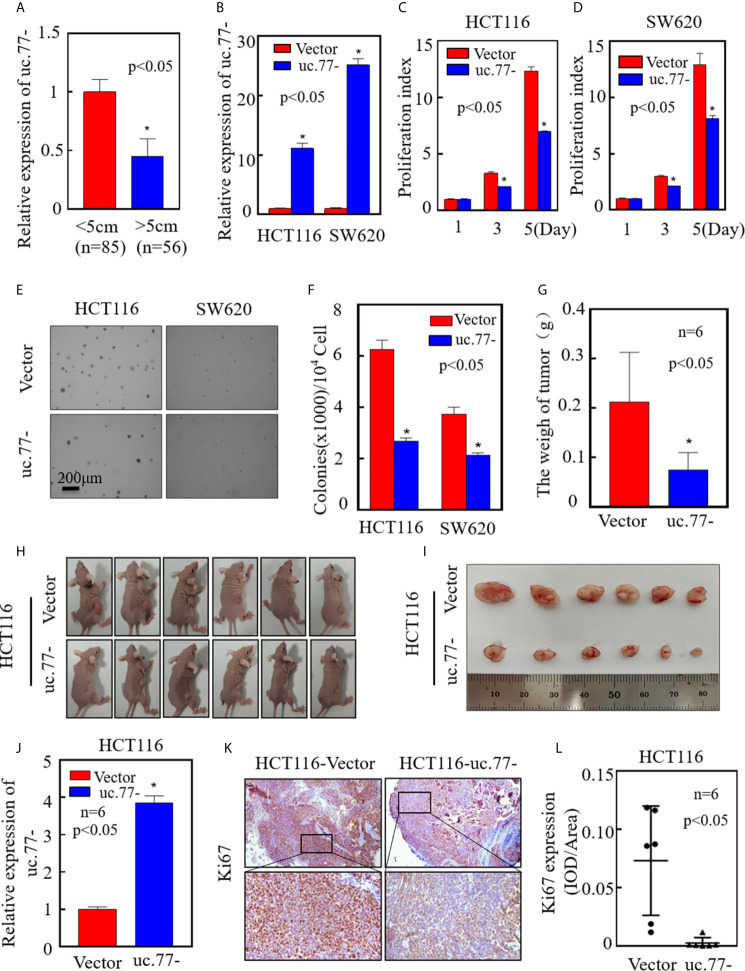
uc.77- promotes the growth of CRC cells *in vivo* and *in vitro*. **(A).** Real-time PCR was used to detect the expression of uc.77-in fresh clinical cancer tissues. **(B)** Detection of uc.77- stable overexpression by real-time PCR. **(C, D)** Effect of uc.77- on the proliferation of HCT116 **(C)** and SW620 **(D)** cells detected by ATP assays. **(E, F)** Effect of uc.77- on anchorage-independent growth in HCT116 and SW620 cells detected by soft agar assays. **(E)** Representative microscope images and **(F)** number of colonies per 10^4^ cells. **(G–I)** HCT116 (uc.77-) and control vector cells were injected into the right lower abdomen of athymic nude mice. After 3–4 weeks, the mice were euthanized, and the tumors were surgically removed, weighed, and photographed. **(J)**Total RNA was extracted from mouse exnograft tumor tissue, and real-time PCR was used to detect the expression of uc.77-. **(K)** Representative IHC images showing the expression of Ki67 in tumor tissues of mice injected with HCT116 (uc.77-) and control vector cells. **(L)** Optical density of Ki67 IHC staining in mouse tumor tissues. Data are presented as the mean ± SD and analyzed by the Student’s t-test, *P < 0.05.

To further investigate the role of uc.77- in the development of human CRC, real-time PCR was performed to detect the relationship between uc.77- and tumor size. As shown in **Figure 2A**, the uc.77- level was lower in big size tumor than in small size tumor. To ensure the biological function of uc.77- in CRC, an uc.77- overexpression plasmid was constructed and stably transfected into HCT116 and SW620 cells, and the overexpression efficiency was tested (**Figure 2B**). Overexpression of uc.77- inhibited the monolayer proliferation ability of HTC116 and SW620 cells (**Figures 2C, D**). Consistently, the anchorage-independent growth of HTC116 (uc.77-) and SW620 (uc.77-) cells was inhibited compared with that of control vector cells (**Figures 2E, F**). To determine whether uc.77- inhibits the growth of CRC cells *in vivo*, a xenograft tumor nude mouse model was used to examine the effect of uc.77- on the tumorigenicity of HTC116 and SW620 cells. The results showed that overexpression of uc.77- significantly reduced tumor growth compared with that in vector control-injected mice (**Figures 2G–I**). The expression of uc.77- in xenograft tumors was also confirmed by real-time PCR (**Figure 2J**). Immunohistochemical (IHC) staining of xenograft tumor samples was also showed that Ki67 expression was significantly lower in tumor tissues of mice injected with HCT116 (uc.77-) than in control vector tumors (**Figures 2K, L**). Taken together, these results indicated that uc.77- inhibits the growth of CRC cells.

### CDK4 Acts as a Downstream Effector of uc.77- to Regulate CRC Cell Proliferation

**Figure 3:**
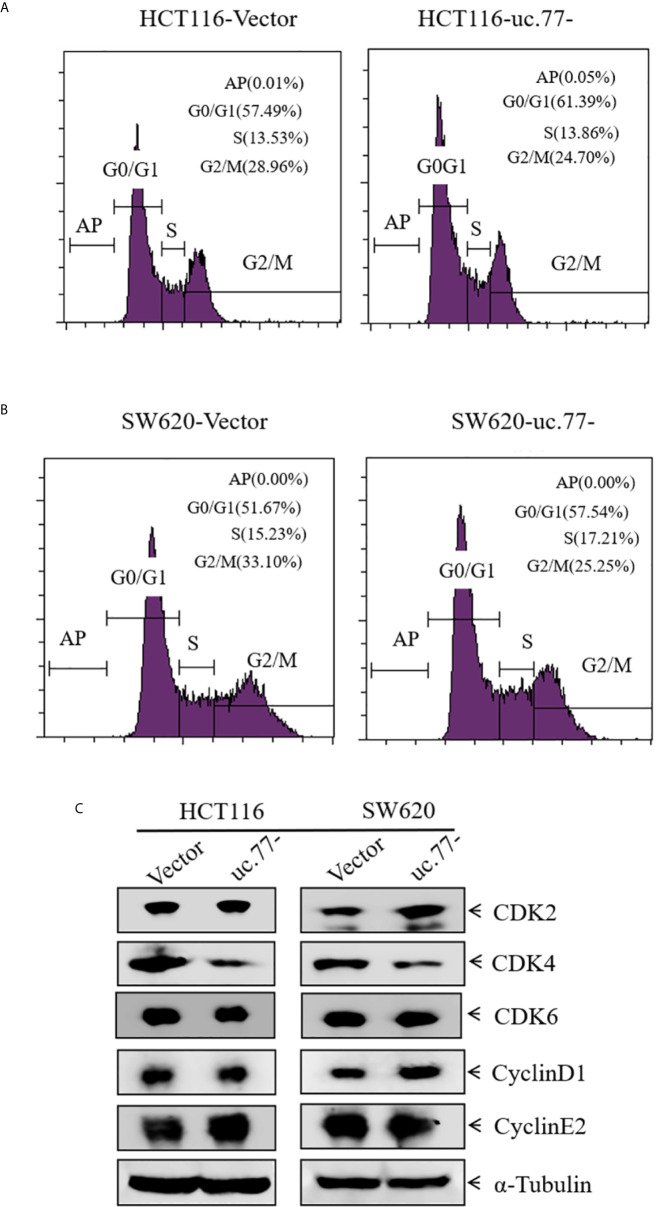
CDK4 acts as a downstream effector of uc.77-. **(A, B)** HCT116 **(A)** and SW620 **(B)** cells as indicated were cultured in 6-well plates, and cell cycle progression was analyzed. **(C)** Expression of CDK2, CDK4, CDK6, cyclin D1, and cyclin E2 in cell lysates detected by western blotting; α-Tubulin was used as a control.

To explore the mechanism underlying the inhibition of CRC cell growth by uc.77-, the effect of uc.77-on cell cycle progression was detected in HCT116 and SW620 cells by flow cytometry. As shown in **Figures 3A, B**, uc.77- caused cell cycle arrest at the G0/G1 phase, suggesting that uc.77- inhibits CRC cell growth by blocking the cell cycle at G0/G1. The effect of uc.77- on G0/G1 cell cycle transition was further examined by assessing the expression of G0/G1 phase-related proteins (CDK2, CDK4, CDK6, cyclin D1, and cyclin E2). The results showed that overexpression of uc.77- downregulated CDK4 in HCT116 and SW620 cells consistently (**Figure 3C**). The results showing that uc.77- inhibits CRC cell proliferation and blocks G0/G1 transition suggested that CDK4 is a downstream effector of uc.77-.

### uc.77- Inhibits CRC Cell Proliferation by Accelerating the Degradation of CDK4

**Figure 4:**
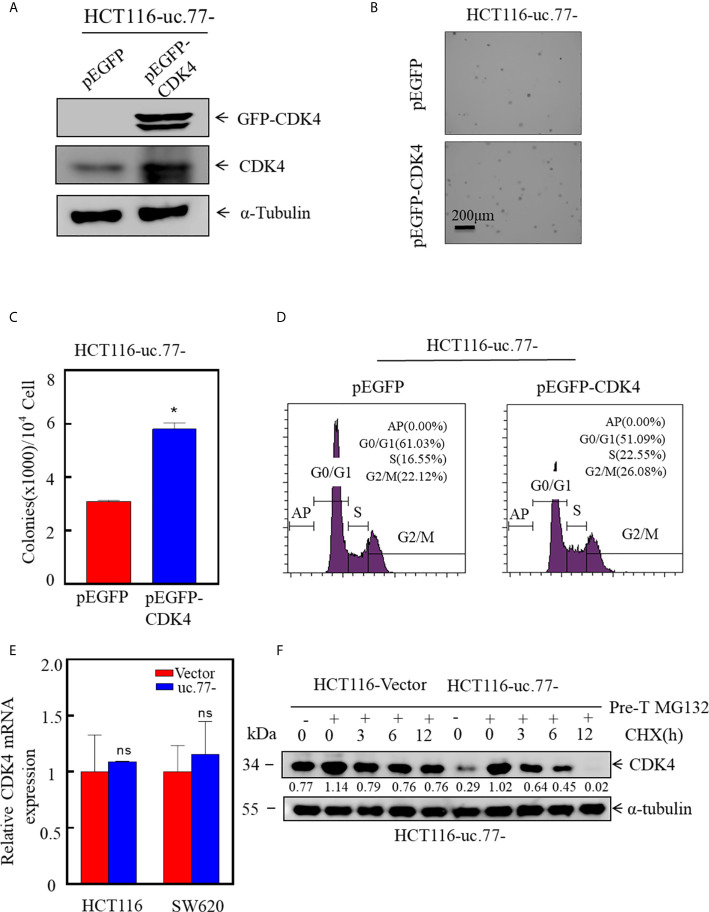
CDK4 promotes the growth of CRC, and uc.77- promotes the degradation of CDK4. **(A)** A CDK4 overexpression vector was stably transfected into HCT116 (uc.77-) cells, and the overexpression efficiency was evaluated by western blotting. **(B, C)** Effect of CDK4 overexpression on the anchorage-independent growth of HCT116 (uc.77-) cells determined by the soft agar assay. **(B)** Representative images and **(C)** number of colonies per 10^4^ cells. **(D)** HCT116 (uc.77-/pEGFP-CDK4) and control cells were cultured in 6-well plates, and cell cycle analysis was performed. **(E)** Detection of CDK4 mRNA expression in HCT116 (uc.77-) and SW620 (uc.77-) cells and the corresponding control vector cells by real-time PCR. **(F)** HCT116 (uc.77-) and control carrier cells were treated with MG132 for 8 h, followed by treatment with CHX for the indicated times. CDK4 degradation was detected by western blotting. Data are presented as the mean ± SD and analyzed by the Student’s *t*-test, *P < 0.05, ns p > 0.05.

To determine whether CDK4 is necessary for the uc.77-mediated inhibition of CRC growth, a CDK4 overexpression plasmid was stably transfected into HCT116 (uc.77-) cells to restore CDK4 expression (**Figure 4A**). The results showed that overexpression of CDK4 rescued the anchorage-independent growth of cells compared with that in HCT116 uc.77-/pEGFP cells (**Figures 4B, C**). Consistently, analysis of the cell cycle showed that overexpression of CDK4 promoted G0/G1 cell cycle transition compared with that in control HCT116 (uc.77-/pEGFP) cells (**Figure 4D**). These data indicate that CDK4 is the downstream effector of uc.77-.

Next, we sought to determine whether uc.77- regulates the expression of CDK4 at the mRNA level or the protein level. First, the effect of uc.77- on the level of CDK4 mRNA was examined by real-time PCR (**Figure 4E**). CDK4 mRNA levels did not differ significantly between uc.77-overexpressing cells and control vector-transfected cells, suggesting that uc.77- did not affect the expression of CDK4 at the mRNA level. We hypothesized that uc.77- modulates the expression of CDK4 at the protein level. Consistent with this hypothesis, the protein level of CDK4 decreased in a time-dependent manner. As shown in **Figure 4F**, the rate of CDK4 protein degradation was faster in uc.77-overexpressing cells than in control plasmid transfected cells, suggesting that uc.77- affects the ubiquitination level of CDK4 by regulating an E3 ligase.

### uc.77- Directly Binds miR-4676-5p and Upregulates FBXW8 in CRC Cells

**Figure 5:**
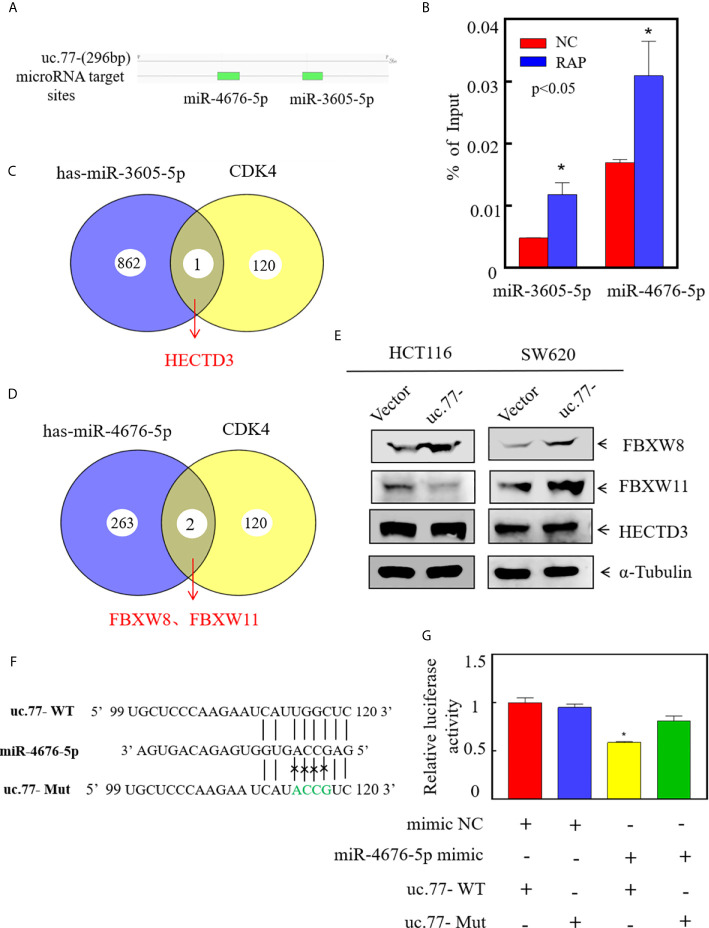
uc.77- binds to miR-4676-5p and indirectly affects downstream CDK4 E3 ligases. **(A)** Potential interacting miRNAs were predicted using (http://regrna2.mbc.nctu.edu.tw/detection.html). **(B)** RNA antisense purification technology was used to detect the binding of uc.77- to selected miRNAs. **(C, D)** E3 ligases were predicted by TargetScan and Ubibrowser. **(E)** FBXW8, FBXW11, and HECTD3 expression was evaluated by western blotting. **(F)** Alignment between miR-4676-5p and the uc.77-seed sequence. WT and Mut represent the wild-type and mutant sequences of uc.77-. **(G)** Luciferase activity of pmirGLO-uc.77–wt and pmirGLO-uc.77–mut upon cotransfection with miRNA mimics NC or miR-4676-5p mimics in 293T cells.Data are presented as the mean ± SD and analyzed by the Student’s *t*-test, *P < 0.05.

To explore the downstream regulatory mechanism of uc.77-, we used RegRNA2.0 (http://regrna2.mbc.nctu.edu.tw/detection.html) to predict potential miRNAs related to uc.77- (**Figure 5A**). RAP experiments indicated that the binding specificity of miR-3605-5p and miR-4676-5p for uc.77- was significantly higher than that of the control sequence probe (**Figure 5B**), which suggested that uc.77- has a binding relationship with miR-3605-5p and miR-4676-5p. Next, we searched for potential E3 ligases using the TargetScan and Ubibrowser databases. The results identified HECTD3 as a potential E3 for miR-3605-5p, and FBXW8 and FBXW11 as potential E3s for miR-4676-5p, indicating that these ligases might affect the degradation of the CDK4 protein (**Figures 5C, D**). Further analysis showed that FBXW8 was the only ligase expressed at higher levels in both HCT116 (uc.77-) and SW620 (uc.77-) cells than in control plasmid transfected cells (**Figure 5E**). These results suggest that FBXW8 acts downstream of miR-4676-5p to affect the degradation of the CDK4 protein. The potential binding sites between uc.77- and miR-4676-5p were analyzed (**Figure 5F**), and a dual luciferase reporter assay was performed to detect the interaction between uc.77- and miR-4676-5p. Co-transfection of uc.77-wild-type vector and miR-4676-5p mimic significantly decreased luciferase activity (**Figure 5G**), indicating that uc.77- interacts directly with miR-4676-5p.

### FBXW8 Is a Direct Target of miR-4676-5p and Mediates CDK4 Protein Stabilization and CRC Cell Growth

**Figure 6:**
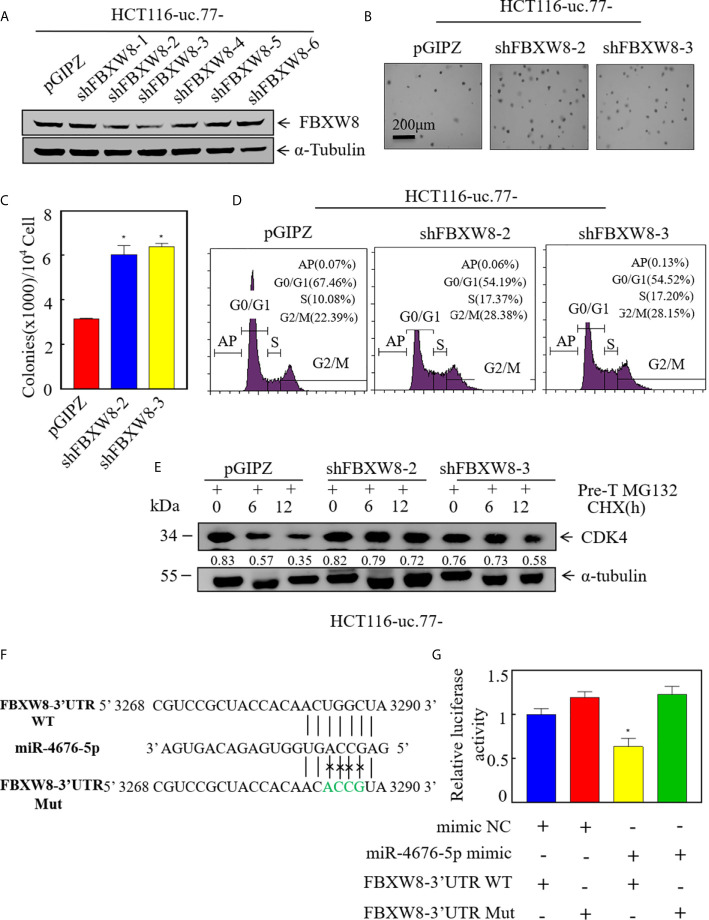
FBXW8 is a direct target of miR-4676-5p, and is involved in CDK4 protein stabilization and CRC cell growth. **(A)** Western blot detection of the knockdown efficiency of FXBW8 in HCT116 (uc.77-) cells. α-Tubulin was used as an internal reference. **(B, C)** Effect of FBXW8 knockdown on anchorage-independent growth detected by soft agar assays; representative microscope images are shown **(B)**, and the number of colonies per 10^4^ cells was counted **(C)**. **(D)** HCT116 (uc.77-/shFBXW8-2), HCT116 (uc.77-/shFBXW8-3), and the corresponding controls were cultured in 6-well plates, and cell cycle analysis was performed. **(E)** HCT116 (uc.77-/shFBXW8-2), HCT116 (uc.77-/shFBXW8-3), and control vector HCT116 (uc.77-/pGIPZ) cells were treated with MG132 for 5 h, and then treated with CHX for the indicated times. CDK4 degradation was detected by western blotting. **(F)** Alignment between miR-4676-5p and the FXBW8 3′-UTR seed sequence. WT and Mut represent wild-type and mutant sequences of the FXBW8 3′-UTR. **(G)** Luciferase activity of pmirGLO-FXBW8 3′-UTR-wt and pmirGLO-FXBW8 3′-UTR-mut upon cotransfection with miRNA mimics NC or miR-4676-5p mimics in 293T cells. Data are presented as the mean ± SD and analyzed by the Student’s *t*-test, *P < 0.05.

To test the role of FBXW8 in mediating the regulatory effect of uc.77- on CRC cell growth, we established stable FBXW8 knockdown HCT116 (uc.77-) cells using a specific shRNA from Open Biosystems (**Figure 6A**). As shown in **Figures 6B–D**, knockdown of FBXW8 significantly reversed the inhibitory effect of uc.77- on HCT116-anchor-independent growth and G0/G1 cell cycle arrest. To examine whether FBXW8 causes CDK4 protein degradation, we performed a protein degradation test. As shown in **Figure 6E**, knockdown of FBXW8 in HCT116 (uc.77-) cells significantly reduced the CDK4 degradation rate. miRNAs bind to the 3′-UTR of a target gene, causing RNA degradation or inhibiting protein translation. IHC staining of xenograft tumor samples was also showed that FBXW8 expression is significantly higher in tumor tissues of mice injected with HCT116 (uc.77-) than in control vector tumors (**Figure S1**). The potential relationship between miR-4676-5p and FBXW8 was examined by analyzing the 3′-UTR of FBXW8 mRNA, including potential miR-4676-5p binding sites (**Figure 6F**). Wild-type and mutant FBXW8 3′-UTR plasmids were co-transfected with miR-4676-5p mimics, and a dual luciferase reporter assay was performed (**Figure 6G**). Mutation of the FBXW8 3′-UTR increased luciferase activity significantly. These results we predicted that miR-4676-5p interacts with the 3′-UTR of FBXW8 mRNA to inhibit FBXW8 protein translation.

## Discussion

CRC is a common malignancy worldwide, and the number of new cases and deaths is increasing. It is estimated that the number of new cases of CRC may reach 2.5 million by 2035 (20). Surgery and chemotherapy are the primary treatments for cancer patients. Biomarkers are important for the detection and treatment of cancer, and the identification of new and sensitive biomarkers is therefore essential (21–23). In this study, we found that uc.77- is downregulated in CRC tissues, and inhibition of CDK4 ubiquitination and degradation mediated by the miR-4676-5p-FBXW8 axis leads to CRC cell growth. These data indicate that uc.77- may serve as a new biomarker and therapeutic target for CRC.

Increasing evidence suggests that T-UCRs play an important role in human diseases, and studies indicate that T-UCRs are involved in the pathogenesis of cancer (24–28). Carlin et al. reported that the expression of T-UCRs is altered in human cancers and that they play vital roles in cancer progression (4). uc.73 and uc.338 are upregulated in CRC and play oncogenic roles in CRC development (4, 5). In the present study, a T-UCR microarray of CRC identified uc.77- as a markedly downregulated T-UCR, suggesting that it could be a potential molecular target in CRC. uc.77 is upregulated in lung cancer cells and plays an oncogenic role in lung cancer by inducing EMT (29). Interestingly, our research found that ZEB2 may not be a key factor in uc77- regulation of human CRC cell proliferation (**Figure S2**). In this study, we demonstrated that uc.77- was significantly downregulated in CRC tissues and cells, and functional experiments *in vivo* and *in vitro* showed that overexpression of uc.77- inhibited the proliferation of CRC cells. This suggests that uc.77- plays a tumor suppressor role in the development of CRC.

The regulatory role of T-UCRs can be mediated by its sponge function through interaction with miRNAs. T-UCRs can bind to miRNAs and decrease the inhibitory effect of miRNAs on the target mRNA, similar to many lncRNAs that interact with miRNAs (30, 31). miRNAs regulate many important physiological functions, and the combination of miRNAs and T-UCRs can alter the function of cells (32). For example, uc.8+ is upregulated in bladder cancer and promotes the development of bladder cancer by interacting with miR-596 (33). uc.173 interacts with pri-miR-195 transcripts to promote the renewal of the intestinal mucosa (34). uc.416+ promotes the development of gastric cancer by interacting with miR-153 (35). uc.339 is highly expressed in non-small cell lung cancer and acts as a sponge for miR-339-3p, miR-663b-3p, and miR-95-5p, which upregulates cyclin E2, the common target of the three miRNAs, thereby promoting cancer growth (36). In this study, we identified the potential miRNA of uc.77- using the RegRNA2.0 software and confirmed it by dual luciferase reporter experiments. The results suggested that uc.77- binds directly to miR-4676-5p. However, a previous study showed that uc.77 regulates ZEB2 in human lung cancer (29). To the best of our knowledge, this study is the first to show that miR-4676-5p acts as an oncogene in CRC. miRNAs bind to the 3′-UTR of target genes to regulate translation or stability (37–39). In this study, dual luciferase reporter experiments showed that miR-4676-5p directly binds to the 3′-UTR of FBXW8 to inhibit its expression. These results not only reveal the function and mechanism of miR-4676, but also provide a potential therapeutic target for the treatment of CRC.

The cell cycle is regulated by the activity of cyclins and the chaperone kinases CDKs. CDKs that induce cell division are often active in cancer, and sustained proliferation signals are recognized as malignant tumor markers (40). Pharmacological inhibitors of CDKs have been researched extensively. However, many compounds lack potency or selectivity. Therefore, controlling the cell cycle remains an unmet goal (8, 41). In the present study, we found that uc.77- promoted the ubiquitination of CDK4 by regulating the E3 ubiquitin ligase FBXW8. The present findings indicate that uc.77- may be a target for the treatment of CRC. FBXW8-mediated ubiquitination and degradation of MRFAP1 is important for the regulation of cell cycle progression (30). However, the role of FBXW8 in CRC remains unclear. This study is the first to propose a mechanism underlying the role of FBXW8 in CRC, and to show that inhibition of FBXW8 reduces the formation of CRC cell colonies. The expression and regulation of FBXW8 and CDK4 in clinical tissues need further study.

**Figure 7:**
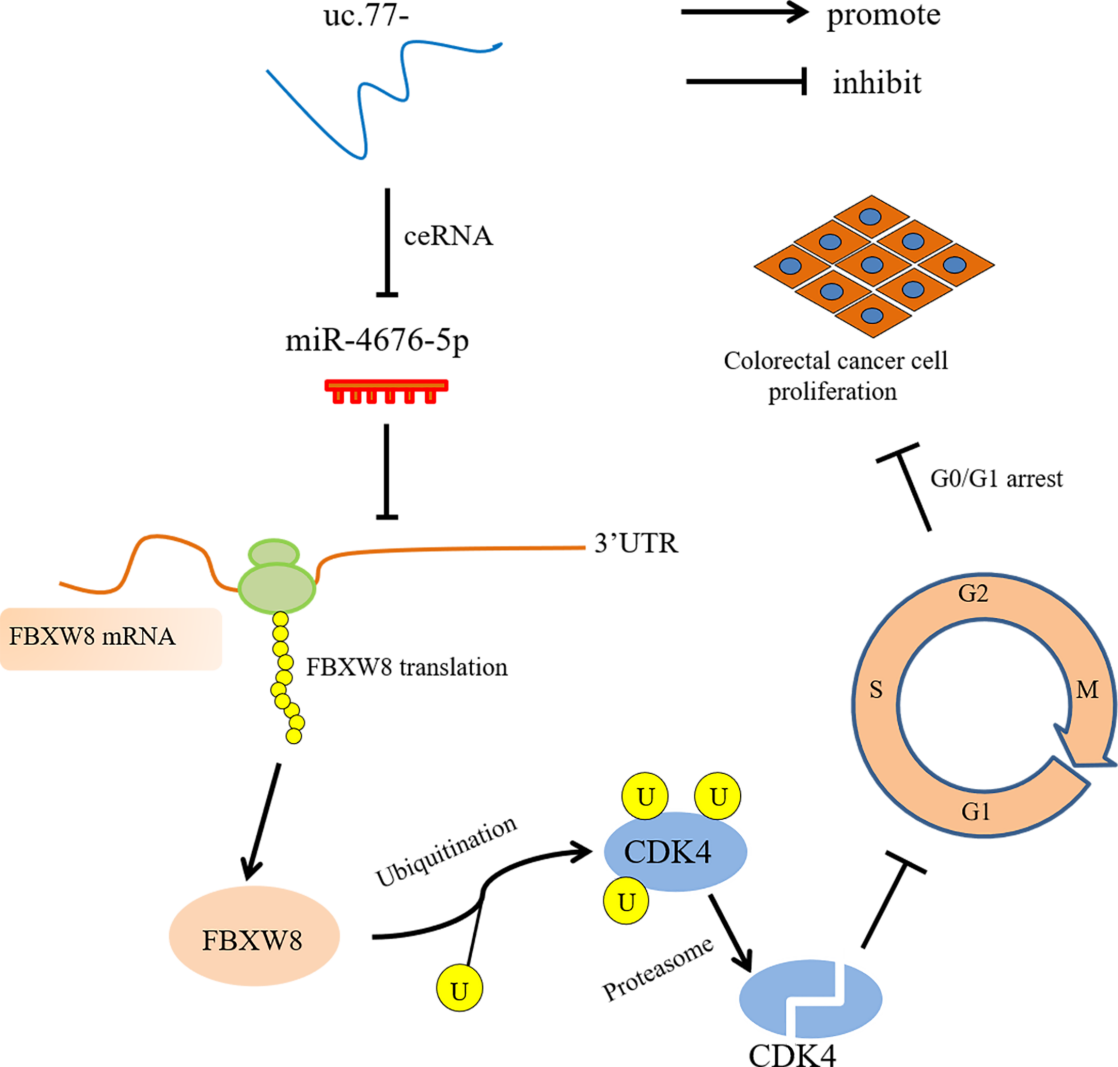
uc.77- schematic diagram of the molecular mechanism underlying the regulation of CRC cell proliferation.

In summary, we showed that uc.77- is downregulated in human CRC and may represent an unfavorable prognostic factor for CRC. Overexpression of uc.77- inhibited the proliferation of CRC cells *in vivo* and *in vitro*. A schematic diagram in **Figure 7** shows that uc.77- competes with FBXW8 to bind miR-4676-5p through a ceRNA mechanism, thereby suppressing the inhibitory effect of miR-4676-5p on the 3′-UTR of FBXW8. The resulting increase in FBXW8 expression and CDK4 ubiquitination results in the downregulation of CDK4 and a block of the G0/G1 transition, thereby inhibiting the proliferation of CRC cells. The findings of this study provide evidence supporting the important role of T-UCRs in CRC, and indicate that uc.77- and its downstream effectors may serve as potential targets for the treatment of CRC.

## Data Availability Statement

The datasets presented in this study can be found in online repositories. The names of the repository/repositories and accession number(s) can be found below: https://www.ncbi.nlm.nih.gov/geo/, GSE167326.

## Ethics Statement

The studies involving human participants were reviewed and approved by Ethics Committee of Wenzhou Medical University. Written informed consent for participation was not required for this study in accordance with the national legislation and the institutional requirements. The animal study was reviewed and approved by Experimental Animal Ethics Committee of Wenzhou Medical University.

## Author Contributions

ZZ, HJ, and HH conceived and designed the study. ZZ, DH, RZ, and XZ detected the cells′ biological function, performed the RT-PCR assays, carried out the soft agar, ATP assay, Western blot, and luciferase reporter assays, and conducted the statistical analyses. YC, SH, QX, and HL analyzed clinical samples. ZZ, HJ, and HH drafted the manuscript. NS and ZL performed immunohistochemistry assay and the statistical analysis. All authors contributed to the article and approved the submitted version.

## Funding

This work was partially supported by Wenzhou Science and Technology Bureau (Y20190065, Y20190061 and Y20180857), and Xinmiao Talent Program of Zhejiang Province (2019R413035).

## Conflict of Interest

The authors declare that the research was conducted in the absence of any commercial or financial relationships that could be construed as a potential conflict of interest.
