# Supplementary material for: uc.77- Downregulation Promotes Colorectal Cancer Cell Proliferation by Inhibiting FBXW8-Mediated CDK4 Protein Degradation
Source: Front Oncol. 2021 May 19;11:673223. doi: 10.3389/fonc.2021.673223 (PMC8172171; doi:10.3389/fonc.2021.673223)
Supplement: Supplementary file 1 [file DataSheet_1.docx]

Table S1. The clinical information of CRC patients.

| case（NO#） | gender | age | stage | case（NO#） | gender | age | stage |
| --- | --- | --- | --- | --- | --- | --- | --- |
| 1# | female | 34 | Ⅳ | 76# | male | 71 | Ⅲ |
| 2# | female | 52 | Ⅲ | 77# | female | 65 | Ⅲ |
| 3# | female | 69 | Ⅰ | 78# | female | 66 | Ⅲ |
| 4# | male | 47 | Ⅳ | 79# | female | 67 | Ⅱ |
| 5# | male | 60 | Ⅱ | 80# | male | 64 | Ⅱ |
| 6# | female | 69 | Ⅰ | 81# | male | 78 | Ⅱ |
| 7# | male | 62 | Ⅲ | 82# | male | 56 | Ⅱ |
| 8# | male | 79 | Ⅰ | 83# | male | 67 | Ⅲ |
| 9# | male | 77 | Ⅳ | 84# | male | 78 | Ⅰ |
| 10# | male | 74 | Ⅰ | 85# | male | 55 | Ⅲ |
| 11# | female | 55 | Ⅰ | 86# | male | 65 | Ⅲ |
| 12# | male | 20 | Ⅰ | 87# | male | 54 | Ⅲ |
| 13# | male | 52 | Ⅱ | 88# | male | 42 | Ⅲ |
| 14# | female | 47 | Ⅲ | 89# | female | 47 | Ⅰ |
| 15# | female | 61 | Ⅱ | 90# | male | 75 | Ⅱ |
| 16# | male | 59 | Ⅳ | 91# | male | 79 | Ⅰ |
| 17# | male | 68 | Ⅳ | 92# | male | 70 | Ⅰ |
| 18# | male | 80 | Ⅰ | 93# | male | 53 | Ⅳ |
| 19# | male | 53 | Ⅰ | 94# | male | 46 | Ⅰ |
| 20# | male | 69 | Ⅲ | 95# | male | 73 | Ⅲ |
| 21# | male | 51 | Ⅳ | 96# | male | 70 | Ⅲ |
| 22# | male | 63 | Ⅳ | 97# | female | 49 | Ⅰ |
| 23# | female | 64 | Ⅱ | 98# | male | 73 | Ⅰ |
| 24# | female | 47 | Ⅰ | 99# | female | 77 | Ⅱ |
| 25# | female | 61 | Ⅰ | 100# | female | 68 | Ⅰ |
| 26# | female | 55 | Ⅰ | 101# | male | 43 | Ⅱ |
| 27# | female | 49 | Ⅲ | 102# | female | 76 | Ⅱ |
| 28# | male | 64 | Ⅱ | 103# | male | 71 | Ⅳ |
| 29# | male | 71 | Ⅰ | 104# | male | 75 | Ⅲ |
| 30# | female | 81 | Ⅲ | 105# | female | 79 | Ⅱ |
| 31# | female | 44 | Ⅳ | 106# | female | 52 | Ⅱ |
| 32# | male | 57 | Ⅰ | 107# | male | 40 | Ⅱ |
| 33# | male | 66 | Ⅰ | 108# | male | 69 | Ⅰ |
| 34# | female | 65 | Ⅲ | 109# | male | 47 | Ⅱ |
| 35# | male | 76 | Ⅰ | 110# | male | 71 | Ⅳ |
| 36# | female | 66 | Ⅲ | 111# | male | 87 | Ⅳ |
| 37# | female | 52 | Ⅲ | 112# | female | 80 | Ⅲ |
| 38# | male | 67 | Ⅳ | 113# | male | 64 | Ⅱ |
| 39# | female | 68 | Ⅰ | 114# | female | 74 | Ⅳ |
| 40# | male | 58 | Ⅰ | 115# | male | 51 | Ⅳ |
| 41# | male | 65 | Ⅳ | 116# | female | 65 | Ⅲ |
| 42# | female | 65 | Ⅱ | 117# | male | 82 | Ⅱ |
| 43# | male | 64 | Ⅱ | 118# | male | 76 | Ⅱ |
| 44# | male | 73 | Ⅰ | 119# | male | 75 | Ⅳ |
| 45# | male | 70 | Ⅱ | 120# | male | 64 | Ⅳ |
| 46# | female | 69 | Ⅲ | 121# | male | 68 | Ⅱ |
| 47# | male | 74 | Ⅱ | 122# | male | 62 | Ⅰ |
| 48# | male | 71 | Ⅳ | 123# | female | 80 | Ⅲ |
| 49# | female | 67 | Ⅳ | 124# | male | 59 | Ⅰ |
| 50# | male | 60 | Ⅱ | 125# | female | 71 | Ⅰ |
| 51# | female | 70 | Ⅱ | 126# | male | 36 | Ⅲ |
| 52# | male | 65 | Ⅲ | 127# | male | 59 | Ⅲ |
| 53# | male | 66 | Ⅲ | 128# | female | 42 | Ⅲ |
| 54# | female | 67 | Ⅰ | 129# | female | 84 | Ⅲ |
| 55# | female | 50 | Ⅳ | 130# | male | 62 | Ⅳ |
| 56# | female | 67 | Ⅳ | 131# | male | 70 | Ⅲ |
| 57# | male | 71 | Ⅲ | 132# | male | 79 | Ⅱ |
| 58# | female | 31 | Ⅲ | 133# | male | 61 | Ⅲ |
| 59# | female | 77 | Ⅲ | 134# | male | 60 | Ⅰ |
| 60# | male | 61 | Ⅰ | 135# | female | 57 | Ⅱ |
| 61# | male | 69 | Ⅰ | 136# | male | 65 | Ⅱ |
| 62# | male | 72 | Ⅳ | 137# | male | 73 | Ⅲ |
| 63# | female | 58 | Ⅳ | 138# | male | 77 | Ⅱ |
| 64# | female | 64 | Ⅱ | 139# | female | 80 | Ⅲ |
| 65# | female | 63 | Ⅲ | 140# | female | 69 | Ⅰ |
| 66# | male | 59 | Ⅱ | 141# | female | 70 | Ⅳ |
| 67# | female | 81 | Ⅱ | 142# | male | 65 | Ⅳ |
| 68# | male | 46 | Ⅲ | 143# | male | 68 | Ⅱ |
| 69# | female | 63 | Ⅱ | 144# | male | 84 | Ⅱ |
| 70# | male | 74 | Ⅱ | 145# | male | 57 | Ⅰ |
| 71# | male | 52 | Ⅲ | 146# | female | 49 | Ⅱ |
| 72# | female | 76 | Ⅲ | 147# | female | 65 | Ⅲ |
| 73# | male | 59 | Ⅰ | 148# | female | 77 | Ⅰ |
| 74# | male | 64 | Ⅱ | 149# | female | 73 | Ⅲ |
| 75# | male | 71 | Ⅲ | 150# | female | 74 | Ⅲ |

Table S2. Sequences of probes used in the RAP experiment.

| Gene | Probe Sequence |
| --- | --- |
| uc.77- | CTTAAGGTTTTAGATTTTCAACTATGAGGGAGAACTCAGCCAAAGATGCT  TAAATGAGCTGGTGTTAGCTATTCATATTTGTTAATGAACTAGATATGCA  ACAATCTTAAATGAAATTTTTATTTTTATGATAGCCATTTACATGTATAA |
| control | ATGGCGATGGCTGGTTTCCATCAGTTGCTGTTGACT |

Figure S1. The expression of uc77- is positively correlated with FBXW8 in human CRC cells.


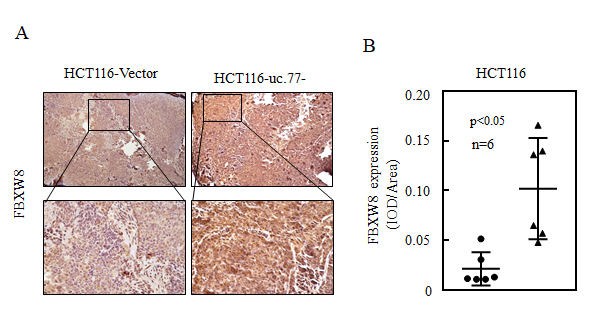


(A) Representative IHC images showing the expression of FBXW8 in tumor tissues of mice injected with HCT116 (uc.77-) and control vector cells. (B) Optical density of FBXW8 IHC staining in mouse tumor tissues.

Supplementary Figure S2. The effects of uc.77- on the expression of ZEB2 in human CRC cells.


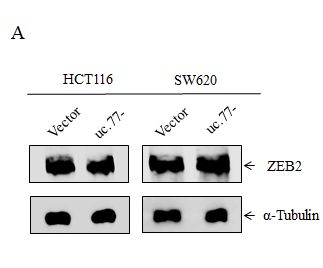


Expression of ZEB2 in cell lysates detected by western blotting; α-Tubulin was used as a control.
